# {4,4′-Dimeth­oxy-2,2′-[2,2-dimethyl­propane-1,3-diylbis(nitrilo­methanylyl­idene)]diphenolato}copper(II) monohydrate

**DOI:** 10.1107/S1600536812038135

**Published:** 2012-09-12

**Authors:** Fatemeh Ganji, Hadi Kargar, Reza Kia, Valiollah Mirkhani, Muhammad Nawaz Tahir

**Affiliations:** a Department of Chemistry, Payame Noor University, PO Box 19395-3697 Tehran, I. R. of IRAN; b Department of Chemistry, Science and Research Branch, Islamic Azad University, Tehran, Iran; c Department of Chemistry, University of Isfahan, 81746-73441, Isfahan, Iran; d Department of Physics, University of Sargodha, Punjab, Pakistan

## Abstract

The asymmetric unit of the title compound, [Cu(C_21_H_24_N_2_O_4_)]·H_2_O, comprises half of a Schiff base complex and a water mol­ecule. The Cu^II^ atom, water mol­ecule and one C atom of the central propyl­ene segment are located on a twofold rotation axis. The geometry around the Cu^II^ atom is distorted square-planar, supported by the N_2_O_2_ donor atoms of the coordinating ligand. The dihedral angle between the symmetry-related benzene rings is 42.56 (19)°. In the crystal, O—H⋯O hydrogen bonds involving the water mol­ecule make an *R*_2_^1^(6) ring motif. Complex mol­ecules are linked into a chain along the *c* axis *via* C—H⋯O inter­actions.

## Related literature

For standard bond lengths, see: Allen *et al.* (1987 ▶). For hydrogen-bond motifs, see: Bernstein *et al.* (1995 ▶). For applications of Schiff bases in coordination chemistry, see, for example: Granovski *et al.* (1993 ▶); Blower *et al.* (1998 ▶). For related structures, see, for example: Ghaemi *et al.* (2011 ▶); Kargar *et al.* (2011 ▶, 2012 ▶).
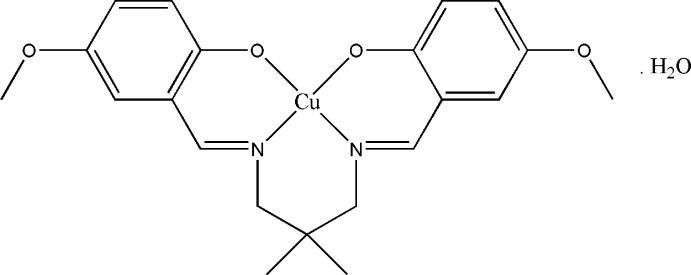


## Experimental

### 

#### Crystal data

[Cu(C_21_H_24_N_2_O_4_)]·H_2_O
*M* *_r_* = 449.98
Orthorhombic, 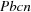

*a* = 20.567 (2) Å
*b* = 12.2647 (14) Å
*c* = 8.4287 (7) Å
*V* = 2126.1 (4) Å^3^
*Z* = 4
Mo *K*α radiation
μ = 1.06 mm^−1^
*T* = 291 K
0.21 × 0.14 × 0.08 mm

#### Data collection

Bruker SMART APEXII CCD area-detector diffractometer
Absorption correction: multi-scan (*SADABS*; Bruker, 2005 ▶) *T*_min_ = 0.256, *T*_max_ = 0.535
17546 measured reflections
2620 independent reflections
1113 reflections with *I* > 2σ(*I*)
*R*_int_ = 0.115

#### Refinement

*R*[*F* ^2^ > 2σ(*F* ^2^)] = 0.051
*wR*(*F* ^2^) = 0.140
*S* = 0.98
2620 reflections
134 parameters
H-atom parameters constrained
Δρ_max_ = 0.28 e Å^−3^
Δρ_min_ = −0.36 e Å^−3^

### 

Data collection: *APEX2* (Bruker, 2005 ▶); cell refinement: *SAINT* (Bruker, 2005 ▶); data reduction: *SAINT*; program(s) used to solve structure: *SHELXTL* (Sheldrick, 2008 ▶); program(s) used to refine structure: *SHELXTL*; molecular graphics: *SHELXTL*; software used to prepare material for publication: *SHELXTL* and *PLATON* (Spek, 2009 ▶).

## Supplementary Material

Crystal structure: contains datablock(s) global, I. DOI: 10.1107/S1600536812038135/kp2436sup1.cif

Structure factors: contains datablock(s) I. DOI: 10.1107/S1600536812038135/kp2436Isup2.hkl

Additional supplementary materials:  crystallographic information; 3D view; checkCIF report

## Figures and Tables

**Table 1:** Selected bond lengths (Å)

N1—Cu1	1.930 (3)
Cu1—O1	1.899 (3)

**Table 2:** Hydrogen-bond geometry (Å, °)

*D*—H⋯*A*	*D*—H	H⋯*A*	*D*⋯*A*	*D*—H⋯*A*
O1*W*—H1*W*⋯O1^i^	0.89	2.43	3.024 (5)	124
C9—H9*A*⋯O1^ii^	0.97	2.59	3.432 (5)	145

Symmetry codes: (i) 
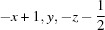
; (ii) 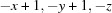.

**Fig. 1.**
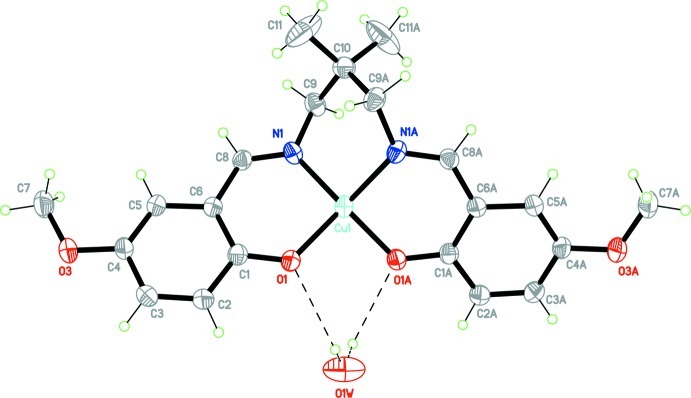
The ORTEP plot of the title compound, showing 40% probability displacement ellipsoids and the atomic numbering.Intermolecular hydrogen bonds between the complex and crystal water molecule are shown (dashed lines).

**Fig. 2.**
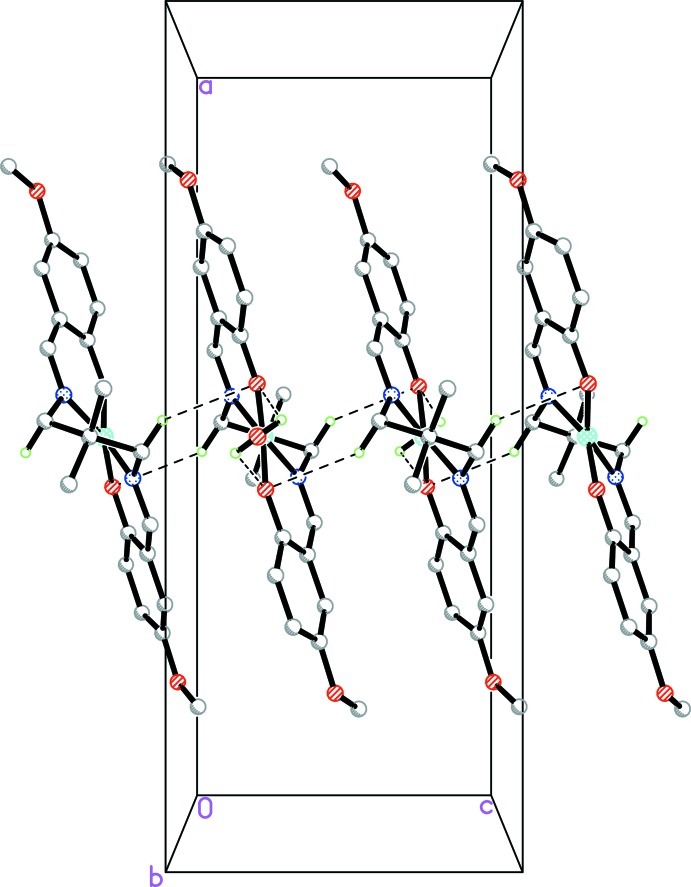
A part of the packing diagram of the title compound showing a chain formed through the intermolecular C—H···O intearctions along the c axis (dashed lines). Only the H atoms involved in the interactions are shown.

## supplementary crystallographic information

### Comment

Schiff base complexes are one of the most important stereochemical models in transition metal coordination chemistry, with the ease of preparation and structural variations (Granovski et al., 1993; Blower et al., (1998). In continuation of our work on the crystal structures of Schiff base metal complexes (Kargar et al., 2012; Kargar et al., 2011; Ghaemi, et al., (2011), we determined the X-ray structure of the title compound.

The asymmetric unit of the title compound (Fig. 1) comprises a a half of Schiff base complex. The bond lengths (Allen et al., 1987) and angles are within the normal ranges and are comparable to the related structure (Kargar et al., 2012; Kargar et al., 2011; Ghaemi, et al., (2011). The geometry around Cu^II^ is a distorted square-planar which is supported by the N_2_O_2_ donor atoms of the coordinated Schiff base ligand (Table 1). The dihedral angle between the substituted benzene rings is 42.56 (19)°. Interamolecular O—H···O hydrogen bonds make R^1^_2_(6) ring motif (Bernstein et al., 1995). The dihedral angle between the symmetry-related benzene rings is 45.54 (19)°. In the crystal structure the molecules are linked together along the c axis, forming a chain through the intermolecular C—H···O interactions (Table 2, Fig. 2).

### Experimental

The title compound was synthesized by adding 5-methoxy-salicylaldehyde-2,2-dimethyl-1, 3-propanediamine (2 mmol) to a solution of CuCl_2_. 4H_2_O (2.1 mmol) in ethanol (30 mL). The mixture was refluxed with stirring for 30 min. The resultant solution was filtered. Dark-green single crystals of the title compound suitable for *X*-ray structure determination were recrystallized from ethanol by slow evaporation of the solvents at room temperature over several days.

### Refinement

The H-atoms were included in calculated positions and treated as riding atoms: C—H = 0.93, 0.96 and 0.97 Å for CH, CH_3_ and CH_2_ H-atoms, respectively, with U_iso_ (H) = k × U_eq_(C), where k = 1.5 for CH_3_ H-atoms, and k = 1.2 for all other H-atoms.

### Crystal data

**Table:**

[Cu(C_21_H_24_N_2_O_4_)]·H_2_O	*F*(000) = 940
*M**_r_* = 449.98	*D*_x_ = 1.406 Mg m^−^^3^
Orthorhombic, *P**b**c**n*	Mo *K*α radiation, λ = 0.71073 Å
Hall symbol: -P 2n 2ab	Cell parameters from 2169 reflections
*a* = 20.567 (2) Å	θ = 2.5–27.4°
*b* = 12.2647 (14) Å	µ = 1.06 mm^−^^1^
*c* = 8.4287 (7) Å	*T* = 291 K
*V* = 2126.1 (4) Å^3^	Block, dark-green
*Z* = 4	0.21 × 0.14 × 0.08 mm

### Data collection

**Table:**

Bruker SMART APEXII CCD area-detector diffractometer	2620 independent reflections
Radiation source: fine-focus sealed tube	1113 reflections with *I* > 2σ(*I*)
Graphite monochromator	*R*_int_ = 0.115
φ and ω scans	θ_max_ = 28.3°, θ_min_ = 3.1°
Absorption correction: multi-scan (*SADABS*; Bruker, 2005)	*h* = −27→27
*T*_min_ = 0.256, *T*_max_ = 0.535	*k* = −16→16
17546 measured reflections	*l* = −10→8

### Refinement

**Table:**

Refinement on *F*^2^	Primary atom site location: structure-invariant direct methods
Least-squares matrix: full	Secondary atom site location: difference Fourier map
*R*[*F*^2^ > 2σ(*F*^2^)] = 0.051	Hydrogen site location: inferred from neighbouring sites
*wR*(*F*^2^) = 0.140	H-atom parameters constrained
*S* = 0.98	*w* = 1/[σ^2^(*F*_o_^2^) + (0.0498*P*)^2^ + 0.1009*P*] where *P* = (*F*_o_^2^ + 2*F*_c_^2^)/3
2620 reflections	(Δ/σ)_max_ < 0.001
134 parameters	Δρ_max_ = 0.28 e Å^−^^3^
0 restraints	Δρ_min_ = −0.36 e Å^−^^3^

### Special details

**Table:**

Geometry. All esds (except the esd in the dihedral angle between two l.s. planes) are estimated using the full covariance matrix. The cell esds are taken into account individually in the estimation of esds in distances, angles and torsion angles; correlations between esds in cell parameters are only used when they are defined by crystal symmetry. An approximate (isotropic) treatment of cell esds is used for estimating esds involving l.s. planes.
Refinement. Refinement of F^2^ against ALL reflections. The weighted R-factor wR and goodness of fit S are based on F^2^, conventional R-factors R are based on F, with F set to zero for negative F^2^. The threshold expression of F^2^ > 2sigma(F^2^) is used only for calculating R-factors(gt) etc. and is not relevant to the choice of reflections for refinement. R-factors based on F^2^ are statistically about twice as large as those based on F, and R- factors based on ALL data will be even larger.

### Fractional atomic coordinates and isotropic or equivalent isotropic displacement parameters (Å^2^)

**Table:**

	*x*	*y*	*z*	*U*_iso_*/*U*_eq_	
O1W	0.5000	0.1449 (4)	−0.2500	0.136 (2)	
H1W	0.5199	0.1907	−0.1831	0.205*	
N1	0.44802 (15)	0.5832 (3)	−0.1447 (3)	0.0486 (8)	
C1	0.37450 (19)	0.3777 (3)	−0.1853 (4)	0.0476 (10)	
C2	0.3307 (2)	0.2908 (3)	−0.2041 (5)	0.0616 (12)	
H1	0.3448	0.2271	−0.2531	0.074*	
C3	0.2675 (2)	0.2974 (3)	−0.1520 (5)	0.0642 (12)	
H3	0.2395	0.2389	−0.1682	0.077*	
C4	0.24498 (19)	0.3899 (3)	−0.0757 (5)	0.0547 (11)	
C5	0.28556 (19)	0.4760 (3)	−0.0581 (5)	0.0516 (10)	
H5	0.2703	0.5388	−0.0087	0.062*	
C6	0.35058 (18)	0.4730 (3)	−0.1130 (4)	0.0459 (9)	
C7	0.1550 (2)	0.4783 (4)	0.0455 (6)	0.0796 (15)	
H7A	0.1793	0.4947	0.1399	0.119*	
H7B	0.1104	0.4650	0.0730	0.119*	
H7C	0.1575	0.5389	−0.0264	0.119*	
C8	0.38928 (18)	0.5688 (3)	−0.0937 (4)	0.0480 (10)	
H8	0.3704	0.6265	−0.0390	0.058*	
C9	0.4810 (2)	0.6853 (4)	−0.1061 (5)	0.0596 (12)	
H9A	0.5197	0.6691	−0.0450	0.072*	
H9B	0.4525	0.7290	−0.0400	0.072*	
C10	0.5000	0.7548 (5)	−0.2500	0.0626 (16)	
C11	0.4427 (3)	0.8223 (5)	−0.3009 (7)	0.139 (3)	
H11A	0.4546	0.8661	−0.3906	0.209*	
H11B	0.4295	0.8688	−0.2150	0.209*	
H11C	0.4073	0.7751	−0.3294	0.209*	
Cu1	0.5000	0.47397 (6)	−0.2500	0.0496 (3)	
O1	0.43439 (12)	0.3654 (2)	−0.2370 (3)	0.0530 (7)	
O3	0.18104 (14)	0.3856 (3)	−0.0270 (4)	0.0734 (9)	

### Atomic displacement parameters (Å^2^)

**Table:**

	*U*^11^	*U*^22^	*U*^33^	*U*^12^	*U*^13^	*U*^23^
O1W	0.216 (7)	0.081 (4)	0.113 (5)	0.000	−0.022 (4)	0.000
N1	0.045 (2)	0.060 (2)	0.041 (2)	−0.0107 (18)	0.0028 (15)	−0.0045 (16)
C1	0.048 (3)	0.052 (3)	0.043 (2)	−0.001 (2)	0.0030 (19)	0.0007 (19)
C2	0.058 (3)	0.049 (3)	0.078 (3)	−0.002 (2)	0.010 (2)	−0.009 (2)
C3	0.054 (3)	0.054 (3)	0.085 (3)	−0.011 (2)	0.010 (2)	−0.005 (2)
C4	0.040 (2)	0.059 (3)	0.065 (3)	−0.001 (2)	0.003 (2)	0.004 (2)
C5	0.046 (2)	0.054 (3)	0.054 (2)	0.003 (2)	0.005 (2)	−0.003 (2)
C6	0.043 (2)	0.048 (2)	0.046 (2)	0.000 (2)	−0.0001 (18)	0.000 (2)
C7	0.053 (3)	0.091 (4)	0.095 (4)	−0.008 (3)	0.019 (3)	−0.006 (3)
C8	0.048 (2)	0.050 (3)	0.046 (2)	0.002 (2)	0.002 (2)	−0.0086 (19)
C9	0.056 (3)	0.068 (3)	0.054 (3)	−0.015 (2)	0.003 (2)	−0.008 (2)
C10	0.072 (4)	0.054 (4)	0.062 (4)	0.000	0.006 (4)	0.000
C11	0.178 (7)	0.137 (5)	0.102 (4)	0.106 (5)	0.047 (4)	0.041 (4)
Cu1	0.0406 (4)	0.0587 (5)	0.0494 (4)	0.000	0.0029 (3)	0.000
O1	0.0433 (15)	0.0533 (17)	0.0624 (18)	0.0010 (12)	0.0043 (15)	−0.0051 (14)
O3	0.0435 (17)	0.073 (2)	0.104 (3)	−0.0068 (16)	0.0141 (16)	−0.0039 (18)

### Geometric parameters (Å, º)

**Table:**

O1W—H1W	0.8940	C7—H7A	0.9600
N1—C8	1.295 (4)	C7—H7B	0.9600
N1—C9	1.460 (5)	C7—H7C	0.9600
N1—Cu1	1.930 (3)	C8—H8	0.9300
C1—O1	1.315 (4)	C9—C10	1.533 (5)
C1—C2	1.405 (5)	C9—H9A	0.9691
C1—C6	1.407 (5)	C9—H9B	0.9699
C2—C3	1.374 (5)	C10—C11^i^	1.503 (6)
C2—H1	0.9300	C10—C11	1.503 (6)
C3—C4	1.384 (5)	C10—C9^i^	1.533 (5)
C3—H3	0.9300	C11—H11A	0.9600
C4—C5	1.354 (5)	C11—H11B	0.9600
C4—O3	1.379 (4)	C11—H11C	0.9600
C5—C6	1.415 (5)	Cu1—O1	1.899 (3)
C5—H5	0.9300	Cu1—O1^i^	1.899 (3)
C6—C8	1.429 (5)	Cu1—N1^i^	1.930 (3)
C7—O3	1.398 (5)		
			
C8—N1—C9	118.5 (3)	N1—C8—H8	116.8
C8—N1—Cu1	125.1 (3)	C6—C8—H8	116.8
C9—N1—Cu1	116.1 (3)	N1—C9—C10	114.7 (3)
O1—C1—C2	118.5 (4)	N1—C9—H9A	108.9
O1—C1—C6	124.5 (4)	C10—C9—H9A	108.9
C2—C1—C6	117.0 (4)	N1—C9—H9B	108.8
C3—C2—C1	121.8 (4)	C10—C9—H9B	107.6
C3—C2—H1	119.1	H9A—C9—H9B	107.7
C1—C2—H1	119.1	C11^i^—C10—C11	113.1 (7)
C2—C3—C4	120.8 (4)	C11^i^—C10—C9^i^	109.4 (3)
C2—C3—H3	119.6	C11—C10—C9^i^	106.3 (3)
C4—C3—H3	119.6	C11^i^—C10—C9	106.3 (3)
C5—C4—O3	125.9 (4)	C11—C10—C9	109.4 (3)
C5—C4—C3	119.0 (4)	C9^i^—C10—C9	112.5 (5)
O3—C4—C3	115.2 (4)	C10—C11—H11A	109.5
C4—C5—C6	121.7 (4)	C10—C11—H11B	109.5
C4—C5—H5	119.1	H11A—C11—H11B	109.5
C6—C5—H5	119.1	C10—C11—H11C	109.5
C1—C6—C5	119.6 (4)	H11A—C11—H11C	109.5
C1—C6—C8	122.5 (4)	H11B—C11—H11C	109.5
C5—C6—C8	117.9 (4)	O1—Cu1—O1^i^	90.97 (15)
O3—C7—H7A	109.5	O1—Cu1—N1^i^	155.08 (11)
O3—C7—H7B	109.5	O1^i^—Cu1—N1^i^	93.83 (12)
H7A—C7—H7B	109.5	O1—Cu1—N1	93.83 (12)
O3—C7—H7C	109.5	O1^i^—Cu1—N1	155.08 (11)
H7A—C7—H7C	109.5	N1^i^—Cu1—N1	92.05 (18)
H7B—C7—H7C	109.5	C1—O1—Cu1	127.2 (2)
N1—C8—C6	126.5 (4)	C4—O3—C7	117.6 (3)
			
O1—C1—C2—C3	179.5 (4)	Cu1—N1—C9—C10	68.1 (4)
C6—C1—C2—C3	−1.1 (6)	N1—C9—C10—C11^i^	−153.9 (4)
C1—C2—C3—C4	−1.3 (7)	N1—C9—C10—C11	83.7 (5)
C2—C3—C4—C5	2.4 (7)	N1—C9—C10—C9^i^	−34.2 (2)
C2—C3—C4—O3	−179.0 (4)	C8—N1—Cu1—O1	−0.3 (3)
O3—C4—C5—C6	−179.6 (4)	C9—N1—Cu1—O1	172.8 (3)
C3—C4—C5—C6	−1.2 (6)	C8—N1—Cu1—O1^i^	−100.9 (4)
O1—C1—C6—C5	−178.4 (3)	C9—N1—Cu1—O1^i^	72.2 (4)
C2—C1—C6—C5	2.2 (6)	C8—N1—Cu1—N1^i^	155.4 (4)
O1—C1—C6—C8	2.4 (6)	C9—N1—Cu1—N1^i^	−31.4 (2)
C2—C1—C6—C8	−177.0 (3)	C2—C1—O1—Cu1	171.9 (3)
C4—C5—C6—C1	−1.1 (6)	C6—C1—O1—Cu1	−7.5 (5)
C4—C5—C6—C8	178.1 (3)	O1^i^—Cu1—O1—C1	161.4 (3)
C9—N1—C8—C6	−176.9 (3)	N1^i^—Cu1—O1—C1	−97.4 (4)
Cu1—N1—C8—C6	−3.9 (5)	N1—Cu1—O1—C1	5.9 (3)
C1—C6—C8—N1	3.7 (6)	C5—C4—O3—C7	0.8 (6)
C5—C6—C8—N1	−175.6 (4)	C3—C4—O3—C7	−177.7 (4)
C8—N1—C9—C10	−118.3 (4)		

Symmetry code: (i) −*x*+1, *y*, −*z*−1/2.

### Hydrogen-bond geometry (Å, º)

**Table:**

*D*—H···*A*	*D*—H	H···*A*	*D*···*A*	*D*—H···*A*
O1*W*—H1*W*···O1^i^	0.89	2.43	3.024 (5)	124
C9—H9*A*···O1^ii^	0.97	2.59	3.432 (5)	145

Symmetry codes: (i) −*x*+1, *y*, −*z*−1/2; (ii) −*x*+1, −*y*+1, −*z*.
